# Parkin regulates translesion DNA synthesis in response to UV radiation

**DOI:** 10.18632/oncotarget.16855

**Published:** 2017-04-05

**Authors:** Xuefei Zhu, Xiaolu Ma, Yingfeng Tu, Min Huang, Hongmei Liu, Fengli Wang, Juanjuan Gong, Jiuqiang Wang, Xiaoling Li, Qian Chen, Hongyan Shen, Shu Zhu, Yun Wang, Yang Liu, Caixia Guo, Tie-Shan Tang

**Affiliations:** ^1^ State Key Laboratory of Membrane Biology, Institute of Zoology, University of Chinese Academy of Sciences, Chinese Academy of Sciences, Beijing 100101, China; ^2^ CAS Key Laboratory of Genomics and Precision Medicine, Beijing Institute of Genomics, University of Chinese Academy of Sciences, Chinese Academy of Sciences, Beijing 100101, China

**Keywords:** Parkin, translesion DNA synthesis, ultraviolet radiation, melanoma, Parkinson's disease

## Abstract

Deficiency of Parkin is a major cause of early-onset Parkinson's disease (PD). Notably, PD patients also exhibit a significantly higher risk in melanoma and other skin tumors, while the mechanism remains largely unknown. In this study, we show that depletion of Parkin causes compromised cell viability and genome stability after ultraviolet (UV) radiation. We demonstrate that Parkin promotes efficient Rad18-dependent proliferating cell nuclear antigen (PCNA) monoubiquitination by facilitating the formation of Replication protein A (RPA)-coated ssDNA upon UV radiation. Furthermore, Parkin is found to physically interact with NBS1 (Nijmegen breakage syndrome 1), and to be required for optimal recruitment of NBS1 and DNA polymerase eta (Polη) to UV-induced damage sites. Consequently, depletion of Parkin leads to increased UV-induced mutagenesis. These findings unveil an important role of Parkin in protecting genome stability through positively regulating translesion DNA synthesis (TLS) upon UV damage, providing a novel mechanistic link between Parkin deficiency and predisposition to skin cancers in PD patients.

## INTRODUCTION

*PARK2* gene, which is frequently found mutated in early-onset Parkinson's disease (PD) [1, 2], encodes an evolutionarily conserved RING-between-RING E3 ubiquitin ligase Parkin [3, 4]. In addition to being associated with the progression of parkinsonism [5–7], Parkin deficiency is also frequently detected in a broad spectrum of tumors and tumor-derived cell lines, including melanoma, glioma, ovarian cancer, cervical cancer, lung cancer, hepatocellular carcinoma, colorectal cancer, and gastric cancer [8–11]. Parkin knockout mice also exhibit higher susceptibility to tumorigenesis [12, 13], suggesting a role of Parkin in suppressing tumorigenesis. Several biological functions of Parkin have been implicated in tumor suppression [9, 10, 13], such as the role as a pivotal mediator of mitophagy [14–19] and the role as a regulator of cell cycle progression [20, 21]. However, more precise mechanism for Parkin's function in preventing carcinogenesis still needs to be elucidated.

Translesion DNA synthesis (TLS) is one mode of DNA damage tolerance, which utilizes specialized TLS polymerases to sustain DNA synthesis when encountering obstacles [22–24]. TLS polymerase eta (Polη) is specifically required for error-free bypass of UV-induced cyclobutane pyrimidine dimers (CPDs) [25]. Inactivation of Polη is highly related to UV-induced mutagenesis and Polη deficiency lead to a variant form of the human genetic disorder xeroderma pigmentosum (XPV) [26], a disease characterized by an early predisposition to skin cancer. TLS pathway is known to be efficiently triggered by replication stress, such as UV, which leads to uncoupling of replicative polymerase and helicase activities [27], and thereby stretches of single-stranded DNA (ssDNA). SsDNA could be rapidly bound by Replication protein A (RPA), which recruits an E3 ligase Rad18 to stalled replication forks to catalyze PCNA monoubiquitination [28]. Emerging evidences show that the monoubiquitinated PCNA (PCNA-mUb) has a higher affinity with TLS polymerases [29–33]. Therefore, PCNA-mUb is believed to play a key role in orchestrating TLS, which is closely related to genome mutagenesis and genome integrity. The monoubiquitination of PCNA is known to be mediated by Rad18 together with E2 enzyme Rad6 after exposure to replication stress [34, 35], or mediated by CRL4^Cdt2^ complex in unperturbed state [36]. Recently, several factors, such as BRCA1 (breast cancer type 1 susceptibility protein) [37], NBS1 (Nijmegen breakage syndrome 1) [38], Chk1(checkpoint kinase 1) [39], SIVA1 [40], Spartan [41], ZBTB1 [42], MSH2 [43], Polη [44], REV1 [45], and MAGE-A4 (melanoma Antigen A4) [46], have been identified to regulate TLS in different ways, indicating that TLS is intricately regulated at multiple steps.

In this study, we discovered that Parkin is required for efficient ssDNA generation after UV radiation. Depletion of Parkin impairs UV-induced RPA foci formation. Parkin physically interacts with NBS1 and promotes NBS1 foci formation after exposure to UV radiation. In line with those, UV-induced PCNA-mUb and Polη recruitment are seriously compromised in Parkin-null cells. These results therefore unravel a novel function of Parkin in positive regulation of TLS, providing a new vision for the connection between Parkin deficiency and human malignancy.

## RESULTS

### Parkin-null cells are hypersensitive to UV radiation

**Figure 1:**
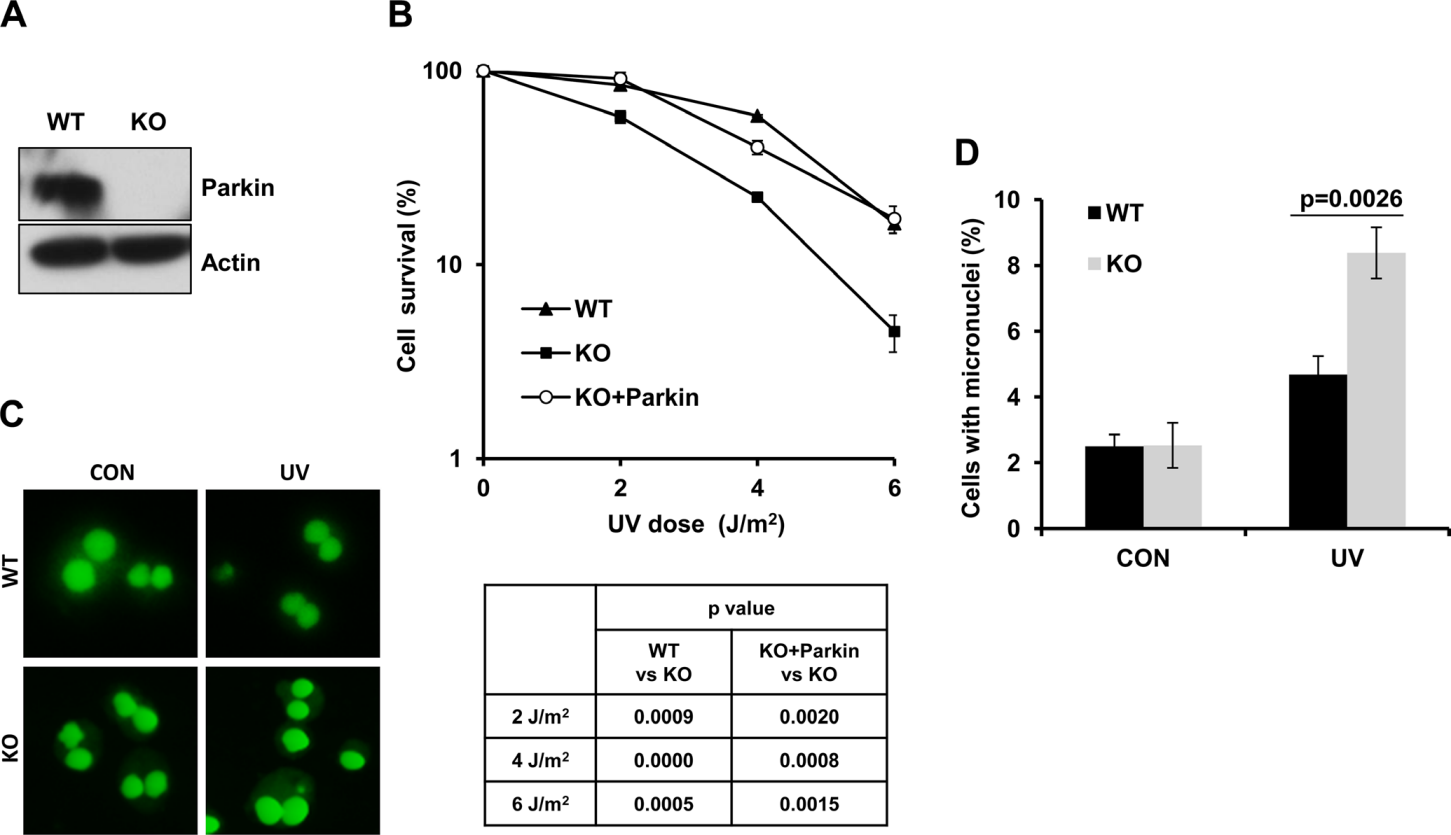
Parkin-null cells are hypersensitive to UV radiation (**A**) Expression of Parkin in WT and Parkin−/− cells were examined by western-blot with an anti-Parkin antibody. β-actin: loading control. (**B**) Viability of cells after exposure to UV radiation. WT, Parkin−/−, and Parkin−/− cells complemented with Parkin were irradiated with the indicated doses of UV, and further incubated in complete medium for 10 days, then cell colonies were determined. Cell viability was expressed as a percentage of mock-treated cells. Error bars represent SD, *t*-test, *n* = 3. (**C**–**D**) WT and Parkin−/− cells were irradiated with 2 J/m^2^ UV, and further incubated in complete medium containing 6 μg/ml CB for 48 h. Then percentage of cells with micronuclei was determined. (C) Representative images of control and UV-irradiated WT and Parkin−/− cells in micronucleus test. (D) Quantification of the percentage of cells with micronuclei. Error bars represent SD. *t*-test, *n* = 3.

PD patients are known to be more susceptible to melanoma [47–49]. Given that the frequency of Parkin mutations or deletions is relatively high in melanoma samples, and Parkin expression usually fails to be detected in melanoma-derived cell lines [8, 50], it is therefore tempting to speculate that Parkin might play an important role in cellular response to UV radiation. To test this possibility, we established wild-type (WT) and Parkin−/− (KO) MEF cell lines (Figure 1A), and tested their viability after exposure to UV radiation through colony assay. Results showed that Parkin-null cells were more sensitive to UV radiation comparing with WT cells (Figure 1B), and complementation with Parkin in Parkin-null cells significantly improved cell viability after UV radiation (Figure 1B), confirming that loss of Parkin was responsible for the hypersensitivity of Parkin-null cells to UV. To further detect the genotoxic effect of UV to WT and Parkin-null cells, we performed a micronucleus test, and found that Parkin-null cells exhibited an increased micronucleus rate after exposure to UV radiation comparing with WT cells (Figure 1C–1D), suggesting that loss of Parkin leads to severe genome instability. All these results collectively indicate that Parkin-null cells are hypersensitive to UV radiation, and Parkin may be important for cellular response to UV-induced DNA damage.

### Parkin promotes PCNA monoubiquitination after UV radiation

**Figure 2:**
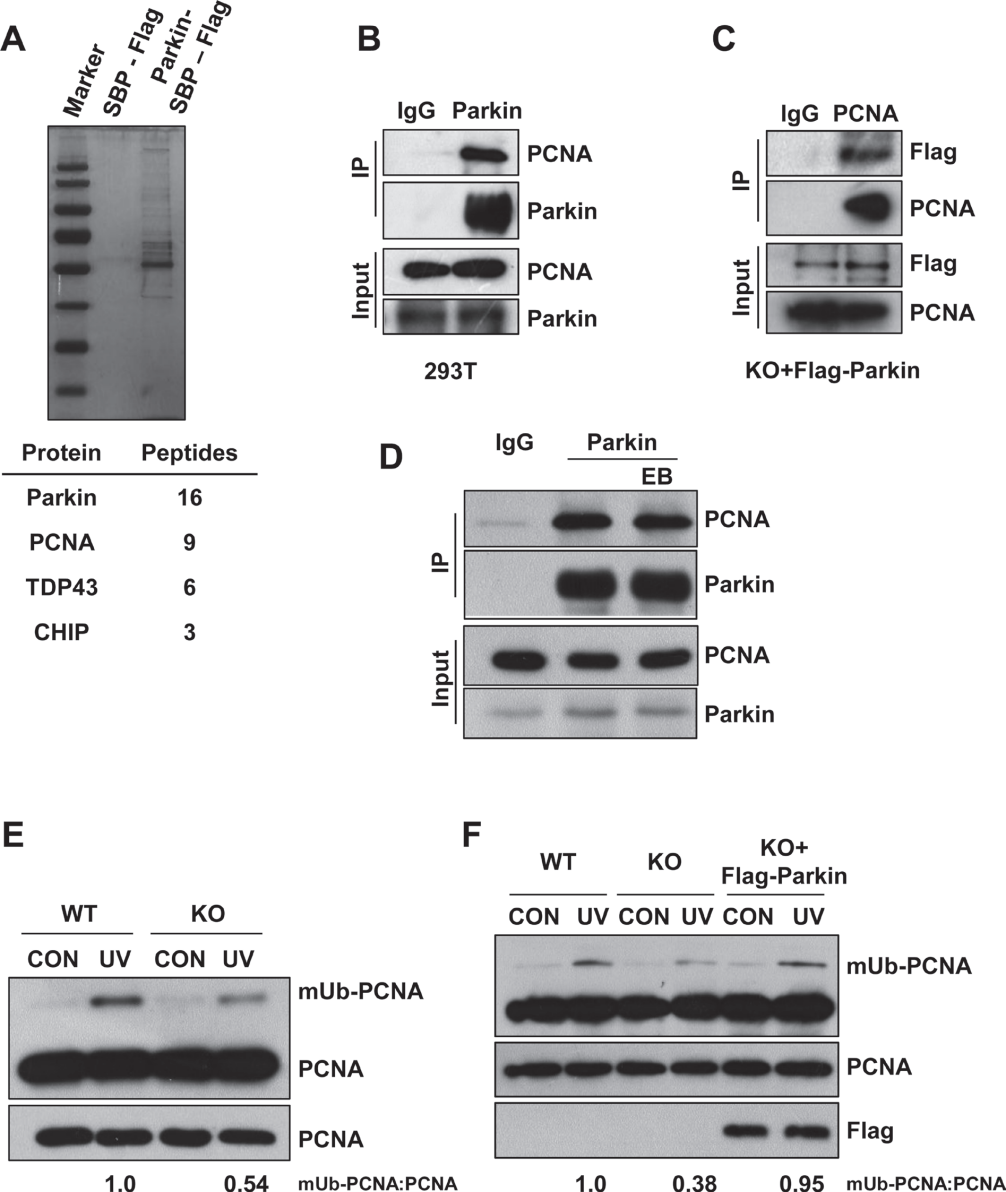
Parkin promotes PCNA monoubiquitination after exposure to UV radiation (**A**) HEK293T cells ectopically expressing empty vector or Parkin-SBP-Flag were used for tandem affinity purification as described in “Materials and Methods”. The final eluates were resolved by SDS-PAGE and revealed by silver staining. Representative proteins identified by mass spectrum were shown below. (**B**) HEK293T cells were lysed and incubated with protein A/G agarose conjugated with normal rabbit IgG or anti-Parkin antibody, the immunoprecipitates were then examined via western blot with antibodies against PCNA and Parkin. (**C**) Parkin−/− cells stably expressing Flag-Parkin were lysed and incubated with protein A/G agarose conjugated with normal mouse IgG or anti-PCNA antibody, the immunoprecipitates were then examined via western blot with antibodies against PCNA and Flag. (**D**) HEK293T cells were lysed and incubated with protein A/G agarose conjugated with normal rabbit IgG or anti-Parkin antibody for immunoprecipitation in the presence of EB or not, followed by blotting with the indicated antibodies. (**E**) WT and Parkin−/− cells were irradiated with 15 J/m^2^ UV and collected 3 h later. The levels of mUb-PCNA were examined by western blot with an anti-PCNA antibody. (**F**) WT, Parkin−/−, and Parkin−/− cells complemented with Flag-Parkin were treated and examined as in (E).

To understand how Parkin functions in cellular response to UV radiation, we performed a tandem affinity purification to identify Parkin-associated proteins. Mass spectrum analysis identified many Parkin associated proteins including PCNA (Figure 2A). To confirm the association between Parkin and PCNA, we performed endogenous immunoprecipitation with an anti-Parkin antibody in HEK293T cells and found that these two proteins interact with each other (Figure 2B), which is in line with a previous report [51]. Meanwhile, we also confirmed the interaction in Parkin-null MEF cells stably expressing Flag-Parkin through immunoprecipitation with an anti-PCNA antibody (Figure 2C). Albeit both Parkin and PCNA could bind to DNA [52, 53], we found that their association was not mediated by DNA (Figure 2D). It is known that PCNA can be monoubiquitinated at K164 after UV radiation [34]. We wondered whether this modification affects the interaction between PCNA and Parkin. We performed half-endogenous immunoprecipitation with an anti-Parkin antibody in HEK293T cells ectopically expressing Flag-PCNA or Flag-PCNA^K164R^, and found that the association between Parkin and PCNA was not affected in the presence of K164R mutation (Supplementary Figure 1). Given that PCNA monoubiquitination is a pivotal step mediating the lesion bypass upon UV radiation and Parkin-null cells exhibit hypersensitivity to UV, we further examined whether Parkin could regulate UV-induced PCNA monoubiquitination. WT and Parkin-null cells were exposed to UV radiation. Interestingly, Parkin-null cells exhibited notably compromised PCNA-mUb in response to UV radiation (Figure 2E). To confirm that Parkin deficiency accounted for the defect, we performed a rescue experiment, and found that complementation with Parkin in Parkin-null cells effectively enhanced PCNA-mUb upon UV radiation (Figure 2F), supporting a role of Parkin in promoting PCNA-mUb in response to UV radiation.

### Parkin promotes Rad18-dependent PCNA monoubiquitination

**Figure 3:**
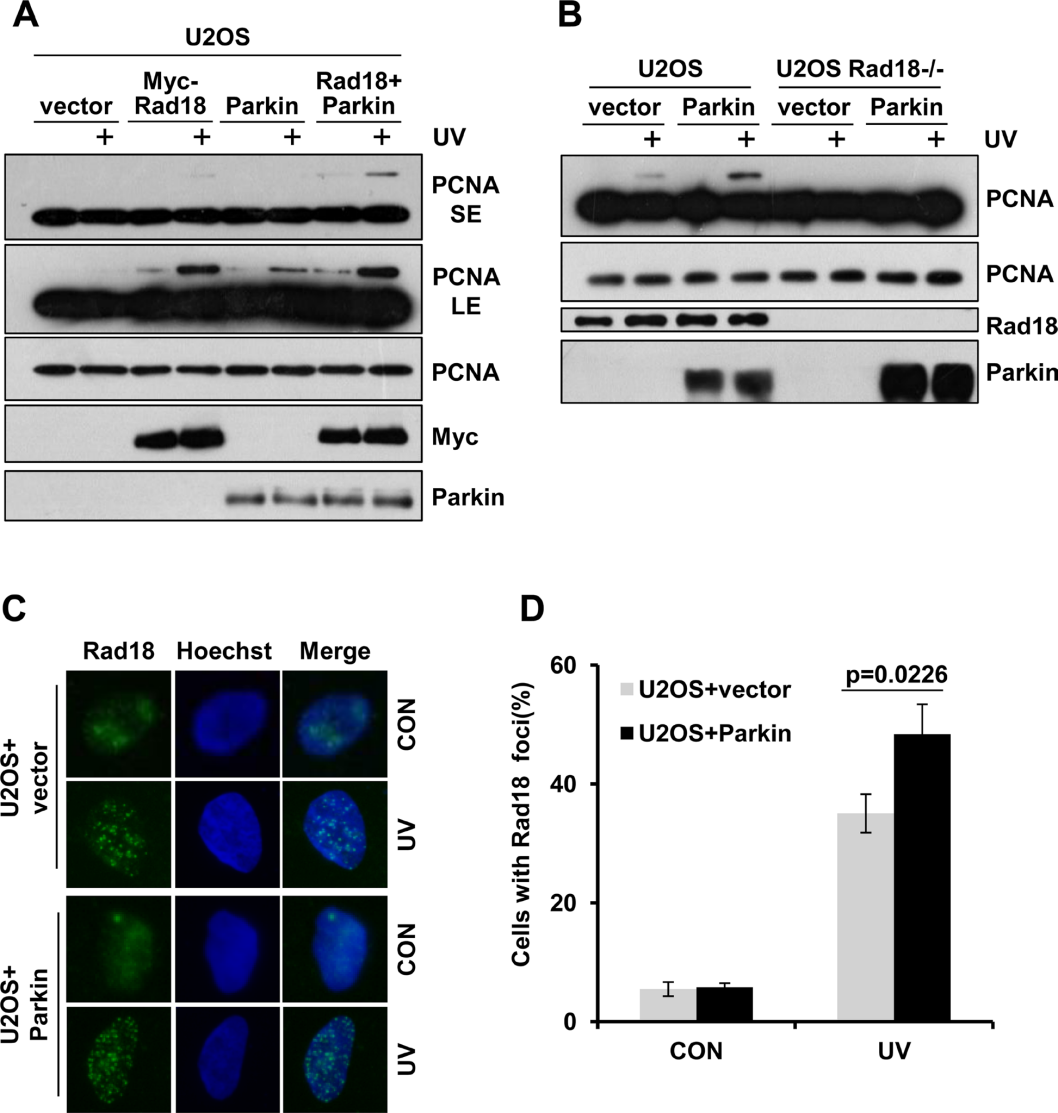
Parkin promotes Rad18 dependent PCNA monoubiquitination (**A**) U2OS cells ectopically expressing Myc-Rad18, Parkin or Myc-Rad18 and Parkin were irradiated with 15 J/m^2^ UV and collected 3 h later. The levels of mUb-PCNA were examined by western blot with an anti-PCNA antibody. SE, short exposure; LE, long exposure. (**B**) WT and Rad18−/− U2OS cells transfected with empty vector or Parkin were treated and examined as in (A). (**C**–**D**) U2OS cells transfected with empty vector or Parkin were irradiated with 15 J/m^2^ UV and recovered for 3 h. Cells were pre-extracted with 0.5% Triton for 10 min and then fixed, immunostained with anti-Rad18 antibody and Hoechst-stained. (C) Representative images of cells stained with Hoechst or antibody against Rad18 after UV radiation. (D) Quantification of the percentage of cells with more than 20 Rad18 foci. Error bars represent SD. *t*-test, *n* = 3.

As Parkin is required for efficient PCNA monoubiquitination, and it is well established that Rad18 mediates the monoubiquitination of PCNA after exposure to UV radiation [35], we wondered whether Parkin regulates this process. U2OS cells ectopically expressing Myc-Rad18, Parkin or Myc-Rad18 and Parkin were collected to examine PCNA-mUb after exposure to UV radiation. Our data showed that overexpression of either Rad18 or Parkin could enhance PCNA monoubiquitination. In addition, ectopic co-expression of Rad18 and Parkin promoted PCNA-mUb to a higher extent comparing with overexpression of Rad18 alone (Figure 3A). Considering the E3 ligase activity of Parkin, it is plausible that Parkin may mediate PCNA-mUb in a Rad18-independent manner. To test this possibility, WT and Rad18-null U2OS cells were transfected with Parkin expression vector and exposed to UV radiation. We found that overexpression of Parkin in WT but not Rad18-null U2OS cells could increase PCNA-mUb level after UV radiation (Figure 3B), indicating that Parkin promotes PCNA-mUb after UV radiation in a Rad18-dependent manner. In line with it, ectopic expression of Parkin enhanced Rad18 recruitment to UV-induced damage sites (Figure 3C–3D).

### Parkin facilitates ssDNA generation and efficient RPA recruitment in response to UV radiation

**Figure 4:**
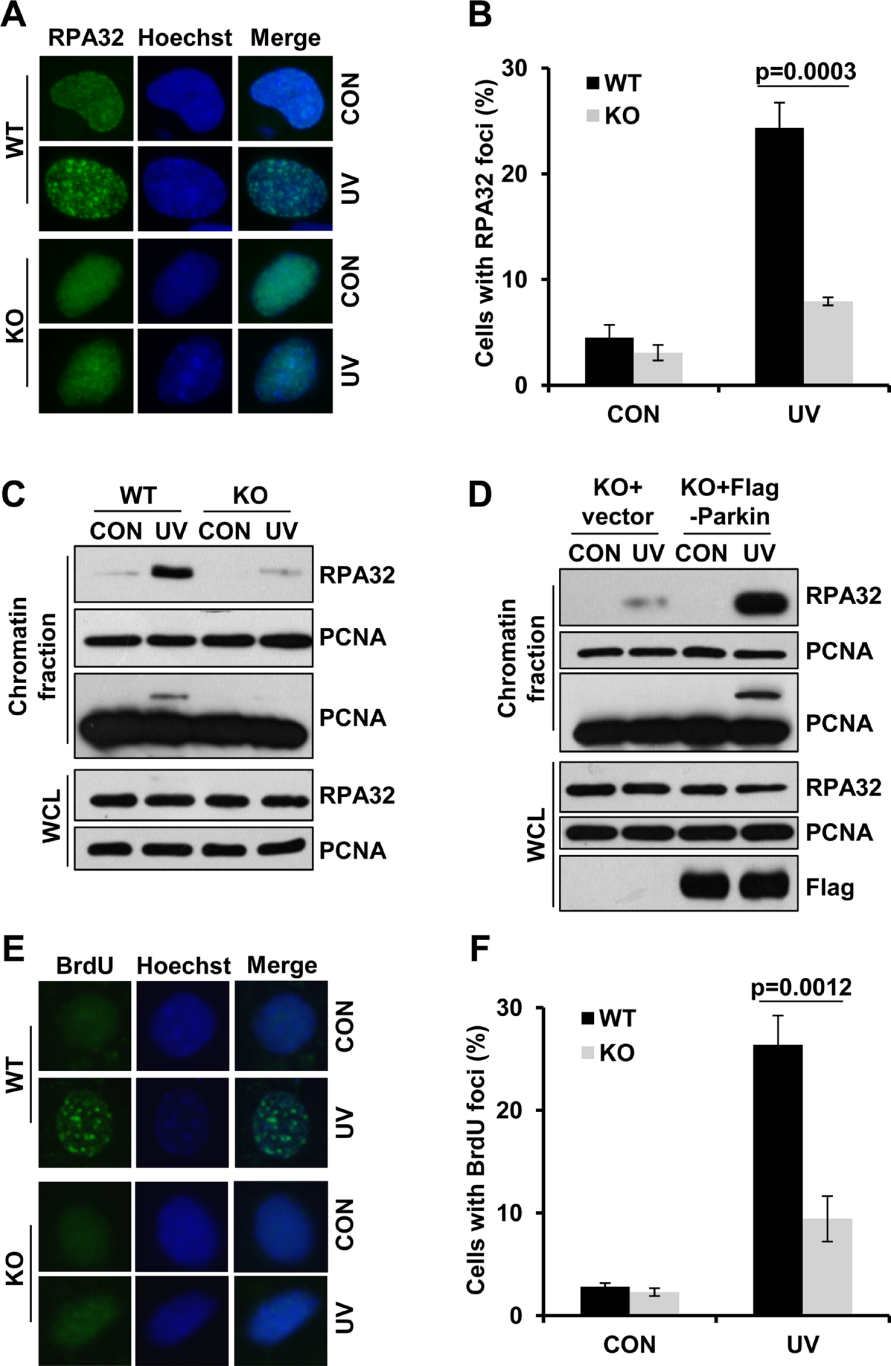
Parkin facilitates ssDNA generation and efficient RPA recruitment in response to UV radiation (**A**–**B**) WT and Parkin−/− cells were irradiated with 15 J/m^2^ UV and further incubated for 2 h. Cells were pre-extracted with 0.5% Triton for 10 min and then fixed, immunostained with anti-RPA32 antibody and Hoechst-stained. (A) Representative images of cells stained with Hoechst or antibody against RPA32 after UV radiation. (B) Quantification of the percentage of cells with more than 20 RPA32 foci. Error bars represent SD. *t*-test, *n* = 3. (**C**) WT and Parkin−/− cells were irradiated with 15 J/m^2^ UV and further incubated for 2 h. The chromatin fraction were harvested and separated by SDS-PAGE. The levels of RPA32 were detected by western blot with an anti-RPA32 antibody. The levels of RPA32 and PCNA in whole-cell lysates (WCL) were also detected. (**D**) Parkin−/− cells complemented with Flag-Parkin or empty vector were treated and examined as in (C). (**E**–**F**) WT and Parkin−/− cells were incubated with 10 μM BrdU for 48 h before irradiated with 15 J/m^2^ UV, and further incubated for 2 h. Cells were pre-extracted with 0.5% Triton and immunostained with anti-BrdU antibody and Hoechst-stained. (E) Representative images of cells stained with Hoechst or antibody against BrdU after UV radiation. (F) Quantification of the percentage of cells with more than 20 BrdU foci. Error bars represent SD. *t*-test, *n* = 3.

Given that Parkin promoted Rad18-mediated PCNA-mUb and Rad18 recruitment after UV radiation, it is likely that Parkin might function at the upstream of Rad18. RPA is a protein reported to facilitate Rad18 recuitment [28]. Here we found that Parkin had a strong association with RPA (Supplementary Figure 2A), and their interaction was dramatically compromised in the presence of EB (Supplementary Figure 2B), indicating that the interaction was largely mediated through DNA. It is known that RPA-ssDNA can serve as a platform for the recruitment of Rad18 to initiate TLS. We further tested whether Parkin was involved in the modulation of RPA recruitment upon UV radiation. WT and Parkin-null cells were exposed to UV radiation, and the percentage of RPA32-foci-positive cells at 2 h post-UV was quantified. Comparing with WT cells, Parkin-null cells exhibited a significant reduction in UV-induced RPA foci formation (Figure 4A–4B). We also compared the chromatin loading of RPA in WT and Parkin-null cells upon UV radiation, and found that the amount of chromatin-associated RPA was remarkably decreased in Parkin-null cells after recovering from UV radiation for 2 h (Figure 4C). To further verify the indispensability of Parkin in optimal UV-induced RPA recruitment, we performed a rescue experiment in Parkin-null cells. We found that complementation with Parkin in Parkin-null cells remarkably enhanced RPA loading after UV radiation (Figure 4D), indicating that Parkin deficiency accounted for the compromised RPA recruitment upon UV radiation.

RPA is known to be recruited to stalled replication forks through its avid affinity with ssDNA. We then determined whether Parkin is required for ssDNA generation upon UV radiation. WT and Parkin-null cells were incubated with 10 μM BrdU for 48 h to label genomic DNA before being irradiated with UV, followed by immunofluorescence with an anti-BrdU antibody without denaturation of DNA. Intriguingly, at 2 h post-UV, Parkin-null cells exhibited considerable decrease in the percentage of BrdU-positive cells comparing with WT cells (Figure 4E–4F), indicating that Parkin facilitates efficient ssDNA generation after exposure to UV.

### Parkin physically interacts with NBS1 and regulates its redistribution after UV radiation

**Figure 5:**
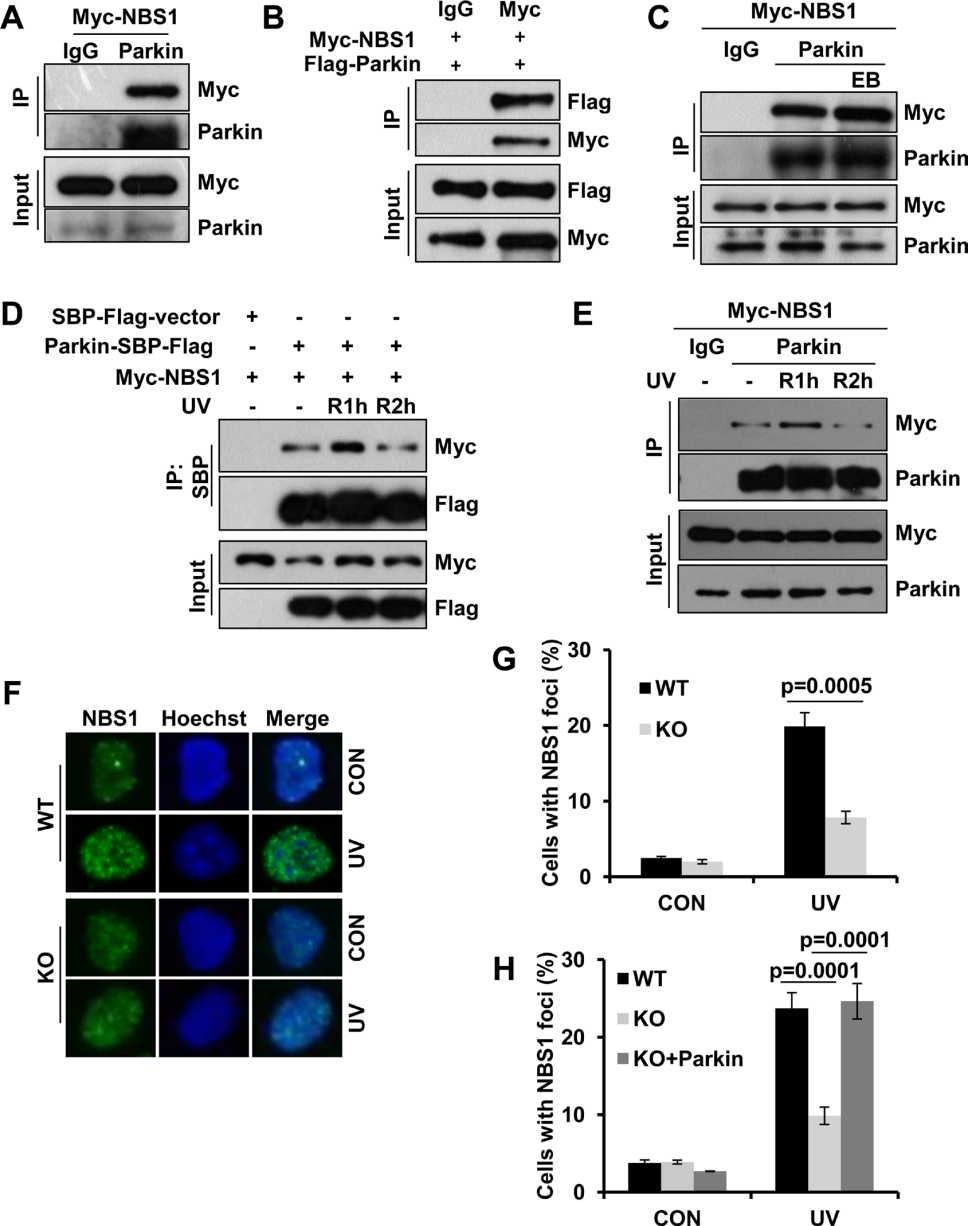
Parkin interacts with NBS1 and regulates its redistribution after exposure to UV radiation (**A**–**B**) Interaction between Parkin and NBS1. HEK293T cells ectopically expressing Myc-NBS1 (A) or Myc-NBS1 and Flag-Parkin (B) were lysed and incubated with protein A/G agarose conjugated with either anti-Parkin or anti-Myc antibody. The immunoprecipitated products were blotted with the indicated antibodies. (**C**) HEK293T cells ectopically expressing Myc-NBS1 were lysed and incubated with protein A/G agarose conjugated with normal rabbit IgG or anti-Parkin antibody for immunoprecipitation in the presence of EB or not, followed by blotting with the indicated antibodies. (**D**–**E**) Dynamic association between Parkin and NBS1 upon UV radiation. (D) HEK293T cells transfected with Myc-NBS1 and Parkin-SBP-Flag or empty vector were irradiated with 15 J/m^2^ UV and recovered for either 1 h (R1h) or 2 h (R2h). Cell lysates were immunoprecipitated with streptavidin-conjugated sepharose followed by blotting with the indicated antibodies. (E) HEK293T cells ectopically expressing Myc-NBS1 were treated as above. Cell lysates were immunoprecipitated with protein A/G agarose conjugated with normal rabbit IgG or anti-Parkin antibody followed by blotting with the indicated antibodies. (F-H) NBS1 foci formation after UV radiation. WT, Parkin−/−, and Parkin−/− cells complemented with Parkin were irradiated with 15 J/m^2^ UV and further incubated for 2 h. Cells were pre-extracted with 0.5% Triton for 10 min and then immunostained with anti-NBS1 antibody and Hoechst-stained. (**F**) Representative images of cells stained with Hoechst or antibody against NBS1 after UV radiation. (**G**–**H**) Quantification of the percentage of cells with more than 20 NBS1 foci. Error bars represent SD. *t*-test, *n* = 3.

In addition to facilitate RPA recruitment, we wondered whether Parkin also regulates UV-induced Rad18 accumulation through other mode(s). Given the key role of NBS1 in RAD18 recruitment after UV radiation [38], we asked whether Parkin interacts with NBS1. Through half-endogenous and exogenous co-IP when either Parkin or NBS1 being immunoprecipated, we discovered that Parkin physically interacted with NBS1 (Figure 5A–5B), and their interaction was not mediated by DNA (Figure 5C). Given that RPA interacts with both NBS1 [54] and Parkin, we wondered whether the association between NBS1 and Parkin is mediated by RPA. We performed immunoprecipitation with an anti-Parkin antibody in WT and RPA32- or RPA70- depleted HEK293T cells ectopically expressing Myc-NBS1, and found that RPA deficiency didn't affect the interaction between Parkin and NBS1 (Supplementary Figure 3). Intriguingly, the Parkin/NBS1 association is under dynamic regulation after UV radiation. The interaction between Parkin and NBS1 was enhanced at 1 h post-UV and decreased thereafter (Figure 5D–5E).

We next examined whether Parkin regulates UV-induced NBS1 foci formation by performing immunofluorescence staining with an anti-NBS1 antibody. WT and Parkin-null cells were exposed to UV radiation, and the percentage of cells with NBS1 foci were quantified at 2 h post-UV. The results showed that albeit the NBS1 protein level of Parkin-null cells is comparable to that of WT cells (Supplementary Figure 4), NBS1 recruitment was significantly reduced in Parkin-null cells comparing with that in WT cells (Figure 5F–5G). We also found that complementation with Parkin in Parkin-null cells significantly enhanced NBS1 recruitment upon UV radiation (Figure 5H), indicating that Parkin promoted NBS1 recruitment to UV-induced damage sites.

We further tested whether the E3 ligase activity of Parkin is essential for its function in the regulation of TLS. We found that complementation with WT Parkin and its E3 ligase inactive mutant Parkin^C431S^ [55] in Parkin-null cells enhanced the recruitment of RPA and NBS1, and PCNA-mUb to a similar extent (Supplementary Figure 5A–5B), and ectopic expression of both Parkin and Parkin^C431S^ in U2OS cells increased the recruitment of Rad18 to UV-induced damage sites (Supplementary Figure 5C), indicating that the E3 ligase acivity of Parkin is dispensable in the role of Parkin in TLS regulation. In line with that, complementation with Parkin^C431S^ in Parkin-null cells also improved cell viability after UV radiation (Supplementary Figure 5D–5E).

### Parkin promotes Polη recruitment to damage sites

**Figure 6:**
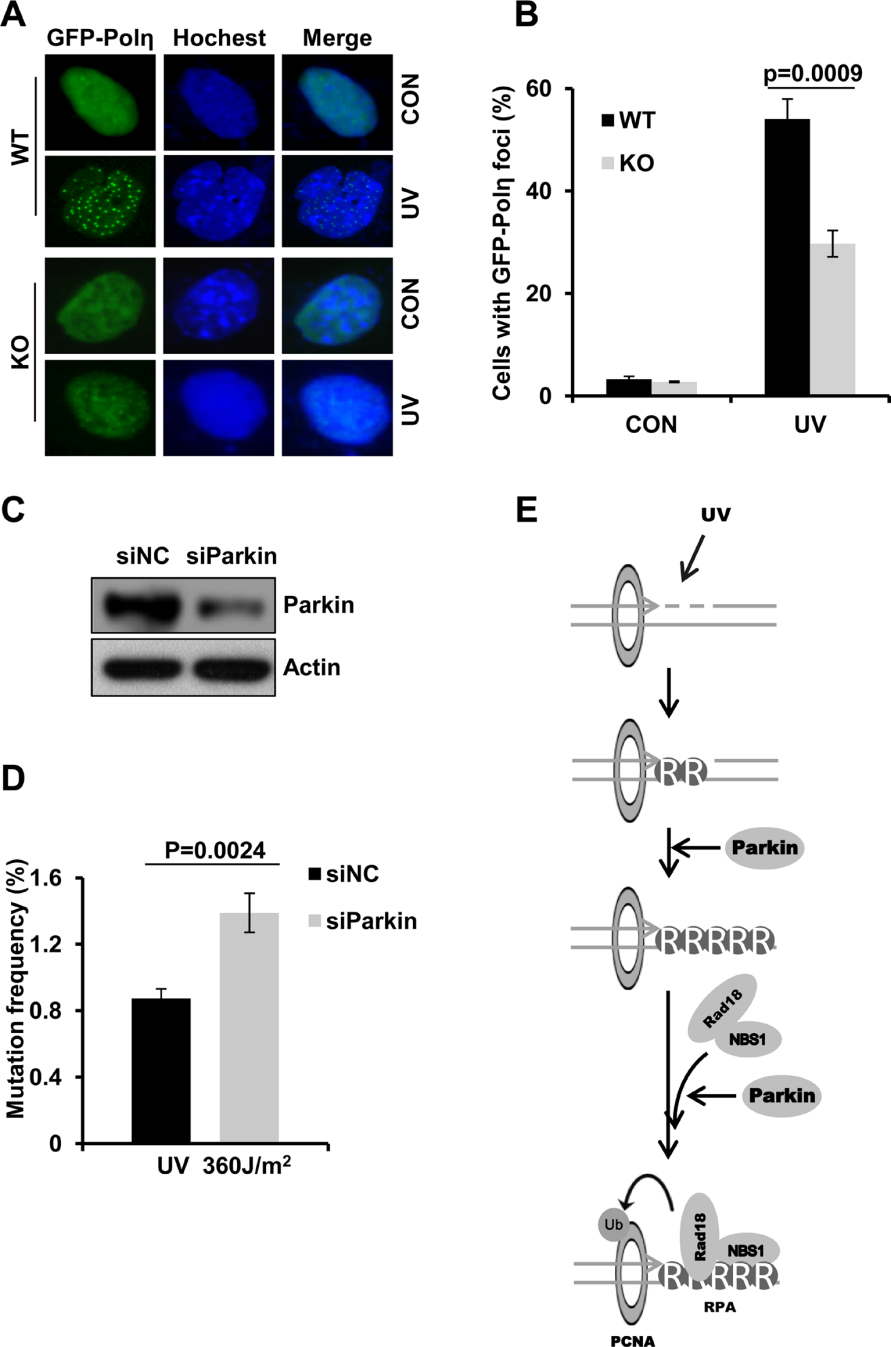
Parkin deficiency attenuates Polη recruitment to damage sites and leads to higher mutation frequency (**A**–**B**) Parkin deficiency was detrimental for recruitment of Polη to UV-induced damage sites. WT or Parkin−/− cells transfected with GFP-Polη were irradiated with 15 J/m^2^ UV and further incubated for 8 h. Then cells were fixed and the proportion of cells with GFP-Polη foci was determined. (A) Representative images of Polη foci formation in WT and Parkin−/− cells. (B) Quantification of the percentage of cells with more than 30 GFP-Polη foci. Error bars represent SD. *t*-test, *n* = 3. (**C**–**D**) Parkin deficiency led to higher mutation frequency. (C) 293T cells were transfected with siParkin or siNC. 72 h later, the cells were harvested and the levels of Parkin were detected by western blot. β-actin: loading control. (D) The Parkin-depleted 293T cells were transfected with pSP189 plasmid which was pre-irradiated with 360 J/m^2^ UV, and replicated for 72 h. The pSP189 plasmid was then extracted and DpnI digested followed by transformation into MBM7070 bacteria strain for mutation frequency analysis. Error bars represent SD. *t*-test, *n* = 3. (**E**) Model for Parkin's function in regulating TLS.

Polη is a TLS polymerase which is recruited to UV-induced sites of damage through its interaction with PCNA-mUb [33]. As Parkin promotes UV-induced PCNA monoubiquitination, we tested whether Parkin depletion compromised Polη recruitment after UV radiation. The percentage of GFP-Polη foci positive cells was quantified and compared in WT and Parkin-null cells irradiated with UV. Our results showed that depletion of Parkin led to dramatically reduced UV-induced Polη foci formation comparing to WT cells (Figure 6A–6B). Recently, Polη was reported to be recruited to sites of oxidative DNA damage through its interaction with PCNA-mUb after laser microirradiation [56], prompted us to ask whether Parkin also regulates this kind of Polη recruitment. WT and Parkin-null cells ectopically expressing GFP-Polη were then exposed to laser microirradiation, and the dynamics of GFP-Polη recruitment to damage sites were monitored. It turned out that Parkin-null cells exhibited attenuated and delayed GFP-Polη recruitment comparing with WT cells (Supplementary Figure 6). These results collectively indicated that Parkin was required for efficient Polη recruitment after DNA damage treatment.

### Parkin deficiency causes increased UV-induced mutagenesis

It is reported that Polη deficiency leads to elevated mutation frequency after UV radiation. Based on the above results that Parkin is required for optimal Polη recruitment to UV-induced damage sites, we speculate that Parkin may also be involved in preventing UV-induced mutagenesis. The mutation frequency was then compared in WT and Parkin-deficient cells using the supF shuttle vector system [46, 57, 58]. UV-irradiated pSP189 plasmids were transfected into control and Parkin-depleted HEK293T cells to replicate. Amplified pSP189 plasmids were extracted 72 h later for mutation frequency measurement. Our results showed that the mutation frequency of UV-damaged pSP189 plasmids in Parkin-depleted cells was higher than that in control cells (Figure 6C–6D), demonstrating an important role of Parkin in preventing UV-induced mutagenesis.

## DISCUSSION

During the past two decades, epidemiologic researches have presented compelling evidences that PD patients exhibit a higher incidence of melanoma [47–49, 59], while the underlying mechanism remains largely unknown. In this study, we unravel a novel function of Parkin, whose deficiency is frequently found in familial PD patients and is also found in sporadic PD patients [5, 7], in TLS regulation after UV radiation. We noticed that Parkin deficiency leads to reduced cell viability and chromosome stability, and increased mutagenesis upon UV radiation, indicating a protective role of Parkin in cellular response to UV radiation.

We found that, although Parkin promotes PCNA-mUb, it failed to facilitate PCNA-mUb in the absence of Rad18. Therefore, it is unlikely that Parkin could mediate PCNA-mUb directly. We also found that Parkin-null cells complemented with both WT and C431S (an E3 ligase inactive form) mutated Parkin exhibited similar levels of RPA and NBS1 recruitment, as well as comparable amounts of PCNA-mUb in response to UV radiation, hinting that the E3 ligase activity of Parkin is not essential for its function in TLS regulation. Based on our results, we propose that Parkin is likely to promote PCNA-mUb by facilitating RPA-ssDNA formation and NBS1 recruitment at UV-induced damage sites (Figure 6E).

It is known that PCNA-mUb is a key factor for optimal recruitment of Polη to stalled replication forks [33], while Polη plays an important role in protecting cells from genome instability after UV radiation [25, 60]. Therefore, the result that Parkin promotes UV-induced Polη foci formation further support the protective role of Parkin in cellular response to UV exposure. Consistently, we noticed that depletion of Parkin increases the mutagenesis after UV treatment, which may explain the phenomena that Parkin deficiency leads to a higher predisposition to UV-induced skin cancer. In line with that, diminished expression of Parkin is frequently found in melanoma samples and melanoma derived cell lines [8]. Deletions and mutations of Parkin are prevalent in both familial and sporadic PD [7, 61, 62]. Some mutations of Parkin are deleterious to its E3 ligase activity, while many mutations retain the E3 ligase activity but exhibit decreased stability and increased propensity to form aggregates [63–65], leading to loss of function of Parkin. Considering the genetic relevance of Parkin with both PD and melanoma, its suppressive role in the pathogenesis of these two seemingly disparate diseases nicely interpret the higher morbidity of melanoma in PD patients.

Besides melanoma, deficiency of Parkin is also found in glioma and a wide spectrum of malignancy, where TLS process happens. Although several biological functions of Parkin [9, 10, 13], such as the role in regulating mitophagy [14–19] and cell cycle progression [20, 21], have been proposed to be important for its tumor suppressor function, regulation of TLS might be another considerable mechanism for Parkin to prevent tumorigenesis. It will be interesting to check whether PD patients have more genome mutation relative to the healthy controls.

There are ample evidences revealing that proper DNA damage response is important in neural development and mature nervous system, and deficiency in DNA repair could lead to a variety of neurologic disorders [66, 67]. Given the function of Parkin in positively regulating TLS, it is intriguing to further investigate whether disrupted TLS pathway due to Parkin deficiency contributes to the neurodegeneration in early-onset PD patients.

In summary, this study unravels a novel function of Parkin in regulating TLS, which explains the high incidence of melanoma and other types of skin cancer in PD patients.

## MATERIALS AND METHODS

### Cell culture and transfection

HEK293T and U2OS cells were obtained from the American Type Culture Collection (Rockville, MD). WT and Parkin−/− MEF cells were derived from 13.5 day-old WT and Parkin−/− [68] C57BL6/J mice embryo (The Jackson laboratory). Rad18−/− U2OS cell line was established as described previously [45]. All cells were cultured in DMEM supplemented with 10% fetal bovine serum. Cell transfection was performed using 1 mg/ml Polyethylenimine, Linear (PEI) or Lipofectamine 2000 Transfection Reagent following the manufacturer's protocol.

### Plasmid constructs

Full-length and mutant of Parkin cDNA were PCR-amplified from Parkin-pIRES-GFP plasmid and subcloned into pcDNA3.1/Zeo(+) or pCMV-2×Flag-SBP (modified from p3×Flag-CMV-14(Sigma)) vector.

Parkin-pIRES-GFP is a kind gift from Dr. Frédéric Checler. Parkin-pcDNA3.1/Zeo(+) is a kind gift from Dr. Konstanze F. Winklhofer. Myc-NBS1 is a kind gift from Dr. David J Chen.

### Antibodies and reagents

Anti-Parkin polyclonal antibody was generated by immunizing rabbit with GST-Parkin (residue 258-407) fusion protein expressed and purified from *E. coli*. Anti-PCNA (sc-56), anti-Myc (sc-40), anti-GFP (sc-8334) antibodies, and Protein A/G Plus-Agarose (sc-2003) were from Santa Cruz Biotechnology, Inc. Anti-PCNA (10205-2-AP) and anti-Myc (10828-1-AP) antibodies were from Proteintech Group, Inc. Anti-Parkin (CST4211) and anti-RPA32 (CST2208) antibodies were from Cell Signaling Technology. Monoclonal anti-Flag M2 antibody (F1804), anti-Flag M2 affinity gel (A2220) and Cytochalasin B (857777) were from Sigma-Aldrich. Anti-Rad18 (ab57447) antibody was from Abcam. Anti-Rad18 (A301-340A) was from Bethyl Laboratories, Inc. Anti-BrdU (347580) antibody was from BD Biosciences. Streptavidin Sepharose (17-5113-01) was from GE Healthcare. PEI (23966) was from Polyscienses, Inc. Lipofectamine® 2000 and Lipofectamine® RNAiMAX Transfection Reagents were from Invitrogen.

### Establishment of stable cell lines with lentivirus infection

cDNAs of full length Parkin/Parkin^C431S^ tagged with Flag were PCR-amplified and subcloned into modified pWPXLd vector (Addgene) containing puromycin resistance. These constructs or pWPXLd empty vector were co-transfected with lentivirus package-plasmid psPAX2 and pMD2.G into HEK293T cells. Lentivirus was collected 36 h later, mixed with equivalent complete medium, and added to cultured Parkin−/− MEFs, incubating for 8 h. Then cells were cultured in fresh medium for 48 h before screening with puromycin for individual stable cell lines.

### RNA interference

The introduction of small interfering RNA (siRNA) into HEK293T cells was carried out with RNAiMAX following the manufacture's protocol. The sequences of siRNAs directed against RPA32 or RPA70 were described previously [69]. ON-TARGET plus Human PARK2 (5071) siRNA-SMART pool was from Dharmacon, Inc.

### Affinity purification of SBP-Flag-tagged Parkin complexes

HEK293T cells were transiently transfected with Parkin-SBP-2×Flag construct or the empty vector. Cells were harvested and lysed with NETN buffer [40], the supernatants were first incubated with anti-Flag M2 agarose for 2 h. The bound proteins were eluted with 200 μg/ml Flag peptide in a buffer with 50 mM Tris-HCl (PH7.4), 150 mM NaCl. Eluted proteins were then incubated with streptavidin-conjugated sepharose for 1 h, bound proteins were eluted with 2 mg/ml Biotin in NETN. Eluent from streptavidin-conjugated sepharose were resolved by SDS-PAGE and revealed by silver staining. The excised gel bands were analyzed with mass spectrometry.

### Cell survival assay

Cell survival assay following UV treatment was performed as described previously [70]. Briefly, cells were seeded into 6 cm dishes (about 200/dish), and incubated for 24 h before irradiated with indicated doses of UV. After treatment, cells were further incubated for 10 days. Then colonies were fixed and quantified. The survival of UV-exposed cells was determined by relating the cloning efficiency to that of an untreated control.

### Micronucleus test

Cells were seeded into 3.5 cm dishes, and incubated for 24 h. Then cells were irradiated with 0 or 2 J/m^2^ UV, followed by culture for 48 h in complete medium containing 6 μg/ml Cytochalasin B. Cells were then collected, rinsed in PBS, and suspended in 0.075 M KCl for 20 min. Then cells were centrifuged at 300 g for 3 min, to remove most of KCl hypotonic solution. Then cells were re-suspended and stained with 0.01% acridine orange. Cells with two nuclei were counted to examine micronuclei rate.

### Mutation frequency

Mutation frequency was measured using the supF shuttle vector system as described previously [71]. HEK293T cells were transfected with negative control or Parkin siRNA. 48 h later, cells were transfected with UV-irradiated (360 J/m^2^) pSP189 reporter plasmid. 72 h later, the pSP189 plasmids were extracted from HEK293T cells and digested with DpnI. Then pSP189 plasmids were transformed into MBM7070 bacteria strain. The transformed MBM7070 were cultured on LB plates containing X-gal, IPTG and ampicillin. The ratio of white (mutant) and blue (wild-type) colonies represent the mutation frequency in the supF coding region. The pSP189 plasmid and MBM7070 strain were gifts from Dr. M. Seidman.

### Preparation of chromatin fraction

Preparation of chromatin fraction was performed as described previously [33] with some modifications. In brief, cells were rinsed with cold PBS, and incubated for 10 min on ice in CSK buffer (10 mM Pipes [pH 6.8], 100 mM NaCl, 300 mM sucrose, 3 mM MgCl_2_, 1 mM EGTA, 0.2% Triton X-100) with protease inhibitor. After rinsed with cold PBS, the cells were collected and centrifuged. The pellets were then incubated with lysis buffer (50 mM HEPES [pH 7.5], 50 mM NaCl, 0.05% SDS, 2 mM MgCl_2_, 10% Glycerol, 0.1% Triton X-100) containing benzonase for overnight, followed by centrifuge to collect the supernatant.

### Immunofluorescence staining

Cells cultured on coverslips were irradiated with 15 J/m^2^ UV, and recovered for indicated time. Cells were rinsed with PBS, and permeabilized with 0.5% Triton buffer (20 mM HEPES [pH7.4], 50 mM NaCl, 300 mM sucrose, 3 mM MgCl_2_) for 10 min before fixation with 4% PFA for 20 min at room temperature. After blocking with 4% BSA for 30 min, cells were incubated with the indicated antibodies for 1 h. After incubation with secondary Alexa Fluor 488-labeled goat-anti-rabbit or goat-anti-rat antibodies (Molecular Probes, Inc), nuclei were stained with Hoechst. Images were analyzed by fluorescent microscopy.

### Immunofluorescent detection of ssDNA

Cells were incubated with 10 μM BrdU for 48 h prior to irradiation with 15 J/m^2^ UV, 2 h later, the cells were stained with an anti-BrdU antibody and counterstained with Hoechst as described above.

## SUPPLEMENTARY MATERIALS FIGURESS


