# Supplementary material for: Parkin regulates translesion DNA synthesis in response to UV radiation
Source: Oncotarget. 2017 Apr 5;8(22):36423–37. doi: 10.18632/oncotarget.16855 (PMC5482665; doi:10.18632/oncotarget.16855)
Supplement: Supplementary file 1 [file oncotarget-08-36423-s001.pdf]

Parkin regulates translesion DNA synthesis in response to UV radiation

Supplementary Materials

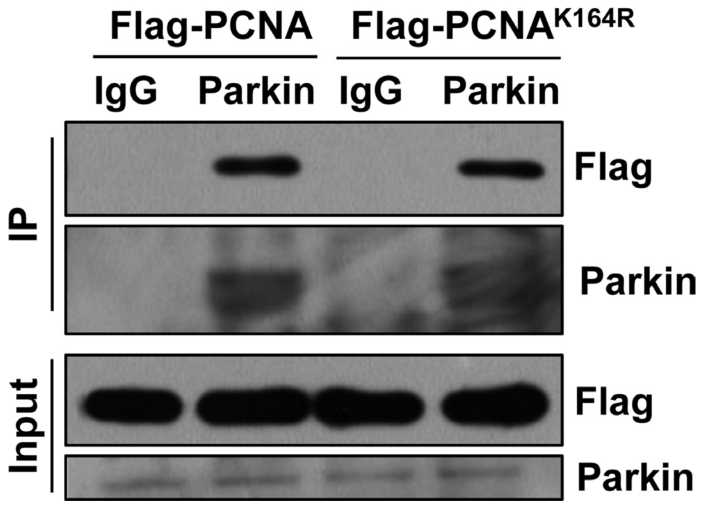

Supplementary Figure 1: K164R mutation doesn't affect the interaction between Parkin and PCNA. HEK293T cells transfected with Flag-PCNA or Flag-PCNA^{K164R} were lysed and incubated with protein A/G agarose conjugated with normal rabbit IgG or anti-Parkin antibody for immunoprecipitation followed by blotting with the indicated antibodies.

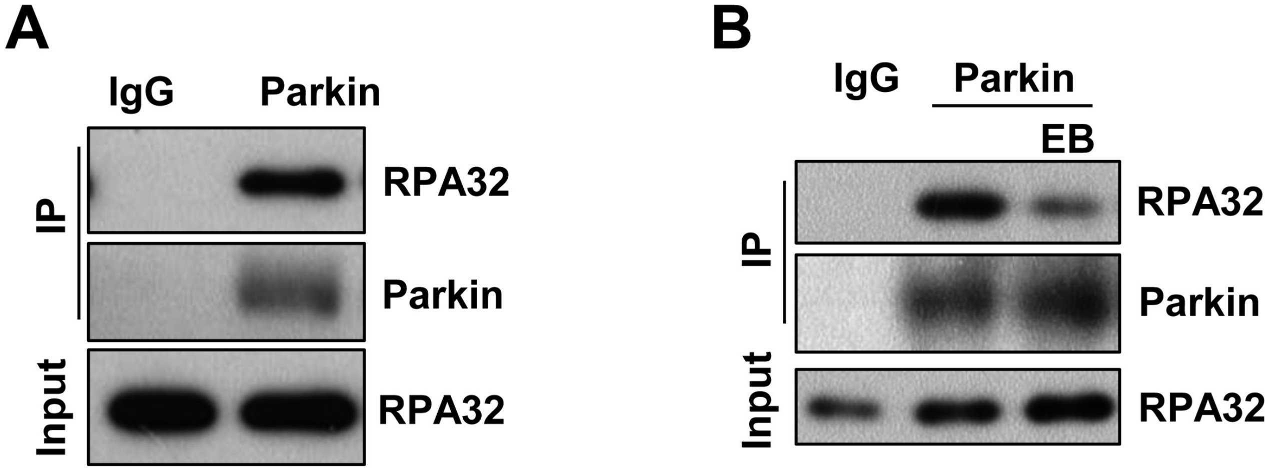

Supplementary Figure 2: Interaction between Parkin and RPA. (**A**) HEK293T cells were lysed and incubated with protein A/G agarose conjugated with normal rabbit IgG or anti-Parkin antibody for immunoprecipitation followed by blotting with the indicated antibodies. (**B**) Endogenous immunoprecipitaiton was performed in HEK293T cell lysates containing EB or not as in (A), followed by blotting with the indicated antibodies.

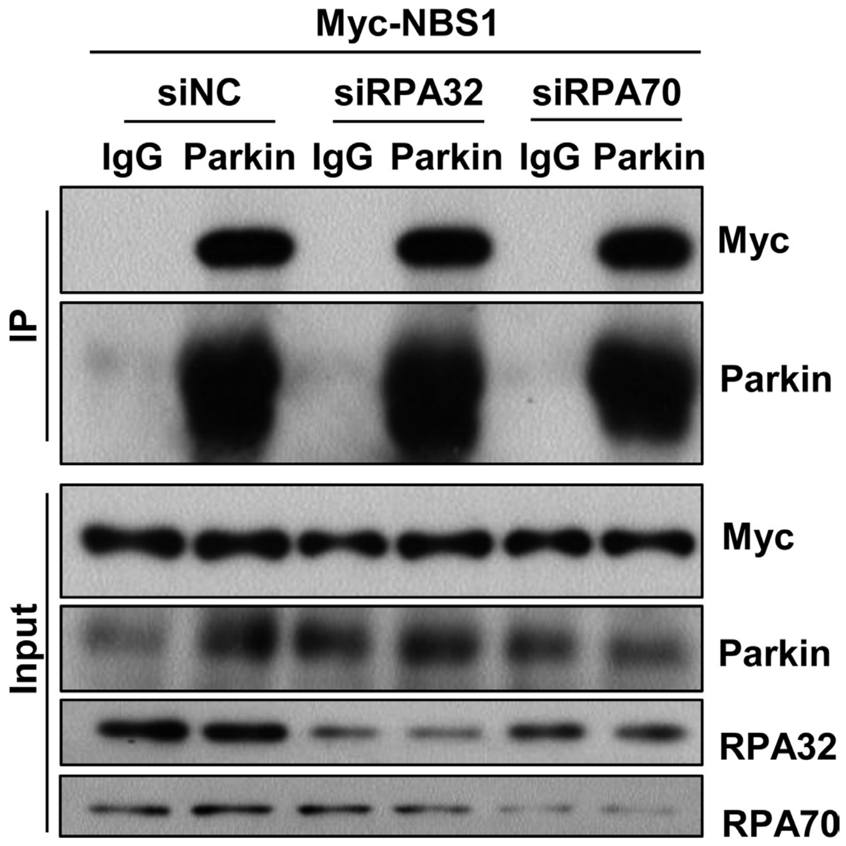

Supplementary Figure 3: The association between Parkin and NBS1 is independent of RPA complex. HEK293T cells were transfected with siNC, siRPA32 or siRPA70. 48 h later cells were transfected with Myc-NBS1. Then cells were lysed and incubated with protein A/G agarose conjugated with normal rabbit IgG or anti-Parkin antibody for immunoprecipitation followed by blotting with the indicated antibodies.

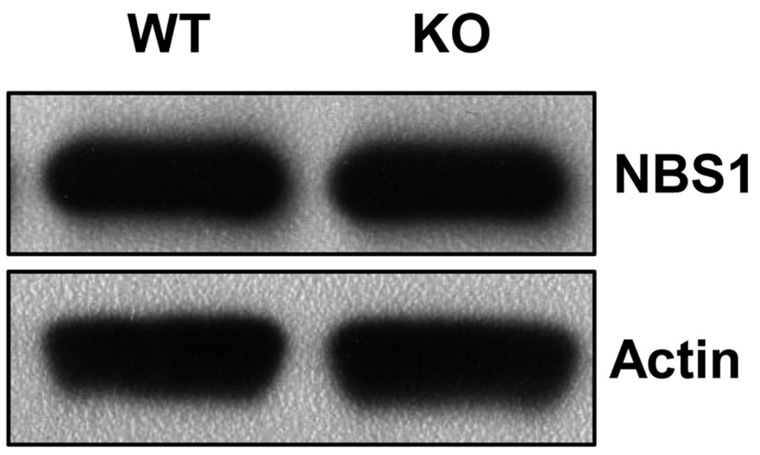

Supplementary Figure 4: Expression of NBS1 in WT and Parkin−/− cells. WT and Parkin−/− cells were collected and examined by western-blot with an anti-NBS1 antibody. β-actin: loading control.

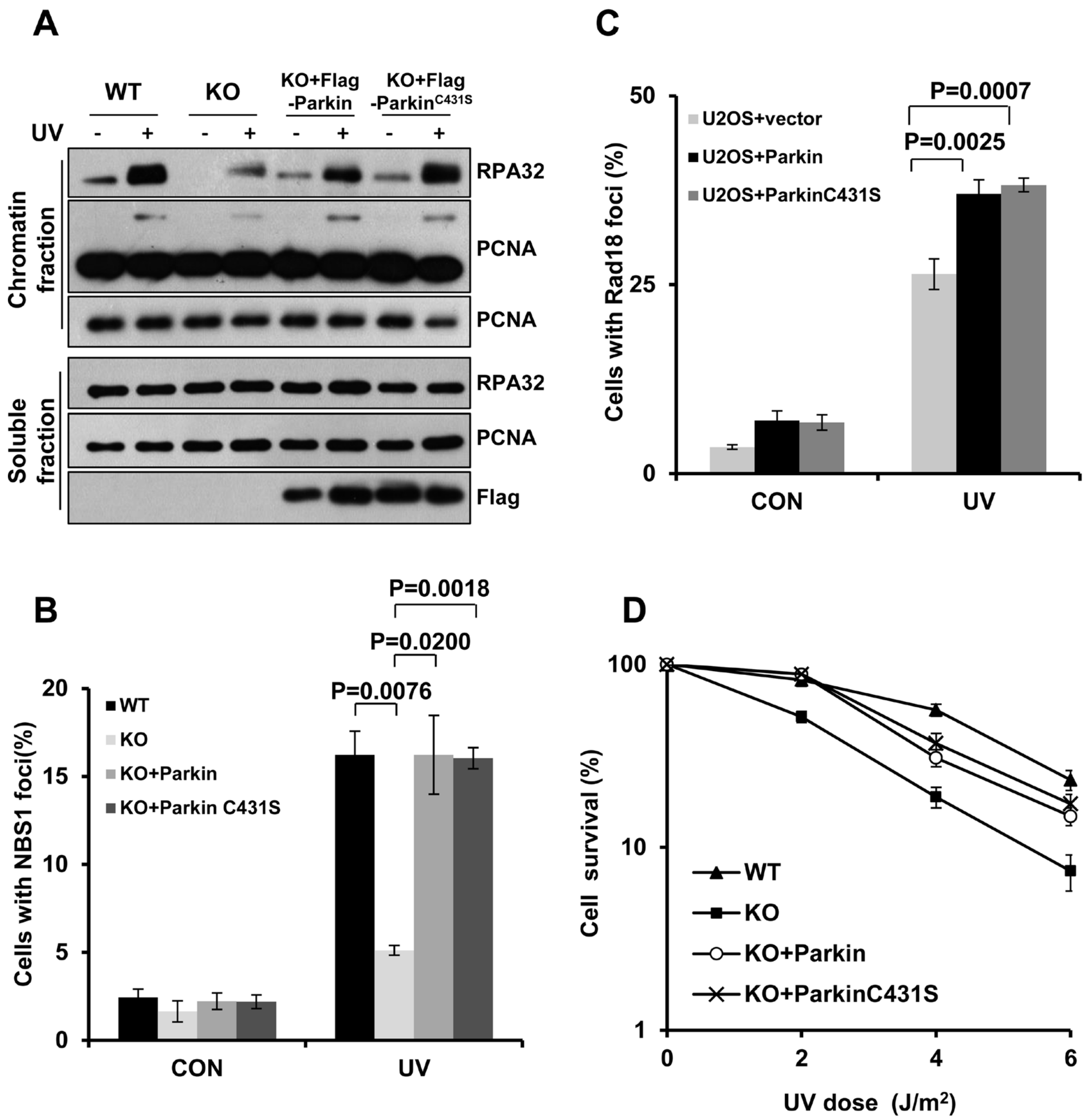

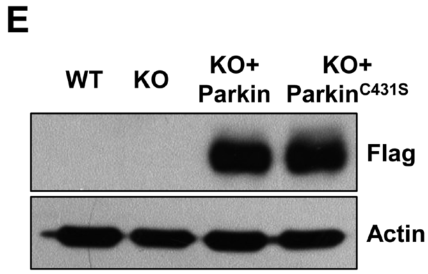

	p value		
	WT vs KO	KO+Parkin vs KO	KO+ Parkin^{C431S} vs KO
2 J/m^2	0.0020	0.0004	0.0006
4 J/m^2	0.0001	0.0072	0.0045
6 J/m^2	0.0011	0.0054	0.0032

Supplementary Figure 5: The function of Parkin in TLS regulation is independent of its E3 ligase activity. (**A**) WT, Parkin−/−, and Parkin−/− cells stably expressing Flag-Parkin or Flag-Parkin^{C431S} were irradiated with 15 J/m^2 UV and further incubated for 2 h. The chromatin fractions were harvested and separated by SDS-PAGE. The levels of RPA32 and mUb-PCNA were detected by blotting with the indicated antibodies. The levels of RPA32 and PCNA in soluble fraction were also detected. (**B**) WT, Parkin−/−, and Parkin−/− cells stably expressing Flag-Parkin or Flag-Parkin^{C431S} were irradiated with 15 J/m^2 UV and further incubated for 2 h. Cells were pre-extracted with 0.5% Triton for 10 min and immunostained with anti-NBS1 antibody and Hoechst-stained. The percentage of cells with NBS1 foci was determined. Error bars represent SD. *t*-test, $n = 2$. (**C**) U2OS cells transfected with empty vector, Parkin or Parkin^{C431S} were irradiated with 15 J/m^2 UV and recovered for 3 h. Cells were pre-extracted with 0.5% Triton for 10 min and then fixed, immunostained with anti-Rad18 antibody and Hoechst-stained. Quantification of the percentage of cells with more than 20 Rad18 foci. Error bars represent SD. *t*-test, $n = 3$. (**D**) Viability of cells after exposure to UV radiation. WT, Parkin−/−, and Parkin−/− cells complemented with Flag-Parkin or Flag-Parkin^{C431S} were irradiated with the indicated doses of UV, and further incubated in complete medium for 10 days, then cell colonies were determined. Cell viability was expressed as a percentage of mock-treated cells. Error bars represent SD, *t*-test, $n = 3$. (**E**) Expression of Flag-Parkin and Flag-Parkin^{C431S} in (D).

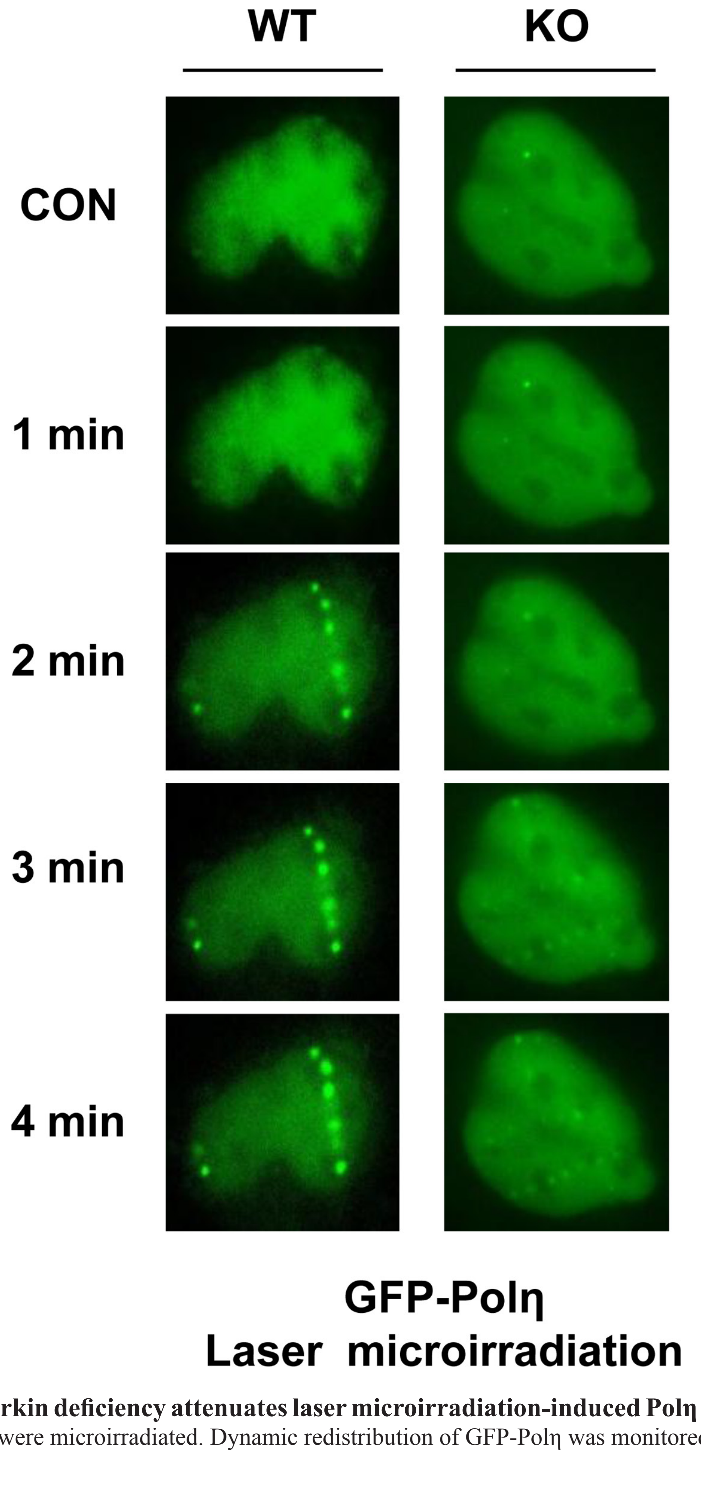

Supplementary Figure 6: Parkin deficiency attenuates laser microirradiation-induced Polη recruitment. WT or Parkin−/− cells transfected with GFP-Polη were microirradiated. Dynamic redistribution of GFP-Polη was monitored at the indicated time points.
